# Supplementary material for: Evidence of a dysregulated vitamin D endocrine system in SARS-CoV-2 infected patient’s lung cells
Source: Sci Rep. 2021 Apr 21;11:8570. doi: 10.1038/s41598-021-87703-z (PMC8060306; doi:10.1038/s41598-021-87703-z)
Supplement: Supplementary file 1 — Supplementary Information 1. [file 41598_2021_87703_MOESM1_ESM.pdf]

SUPPLEMENTARY TABLES AND FIGURE

Evidence of a dysregulated Vitamin D Endocrine System in SARS-CoV-2 infected patient's lung cells

Bijesh George[1, 2], Revikumar Amjesh[1], Aswathy Mary Paul[1, 2], T. R. Santhoshkumar[1], Madhavan Radhakrishna Pillai[3] and Rakesh Kumar[1,4,5]

[1]Cancer Research Program, Rajiv Gandhi Centre for Biotechnology, Trivandrum, INDIA.
[2]PhD Program, Manipal Academy of Higher Education (MAHE), Manipal, INDIA
[3]Pathogen Biology Program, Rajiv Gandhi Centre for Biotechnology, Trivandrum, INDIA.
[4]Department of Medicine, Division of Hematology/Oncology, Rutgers New Jersey Medical School, Newark, USA.
[5]Department of Human and Molecular Genetics, Virginia Commonwealth University, School of Medicine, Richmond, USA.

Supplementary Table 1: The expression profile of Vitamin D components from SARS-CoV2 infected patient samples when compared to its healthy controls and when the analysis performed in A549, NHBE and Calu3 cell lines

Supplementary Table 2: Results of Gene Ontology analysis of 43 genes specifically for COVID19 functional enrichment using Metascape.

Supplementary Table 3: Results of Gene Ontology analysis of 43 using Metascape.

Supplementary Table 4: Functional enrichment analysis of 12 down regulated genes and its network genes using Reactome.

Supplementary Table 5: Functional enrichment analysis of 19 up-regulated genes and its network genes using Reactome.

Supplementary Figure 1: Expression status of 43 share genes identified in Figure 3e in three independent validation datasets from SARS-CoV2 infected patient specimens

	Lung (BALF)	Healthy Lung Biopsy vs COVID19 Lung	SARS2 to Healthy	NHBE Mock vs SARCoV2	A549 Mock vs SARCoV2	A549 Mock vs SARCoV2	Calu3 Mock vs SARCoV2
**Vitamin D component genes**							
RXRA	-2.29						
VDR	-1.85					1.45	0.73
CYP27B1				1.28			
CYP24A1		4.38				0.73	
CYP27A1	-2.57		-2.81				
FGFR1		-3.02	-3.34			-0.96	-0.44
FGFR2	6.76			-0.38		-2.46	-0.54
FGFR3				-0.37		-2.03	-1.64
FGFR4						-2.65	-0.97
* A cut-off of 1.5 foldchange (log2FC .58) is not applied for the above data							
**8 representative molecules**							
ABCB4							
ABCD2							
ACSL1	-0.76	3.47					0.78
ACVR1B	-1.57						
SCNN1a	2.56		4.44			-0.88	
OPN							
CD44	-0.88					0.69	
ITGB3		-4.51		1.21		2.77	
**Vitamin D regulated genes in coagulation**							
PLAU	-2.35					1.70	1.25
PLAT	4.29			1.41			2.46
TFPI	5.24					1.44	0.67
THBD	-4.13			-0.98			2.33
F3	3.70		3.07			1.53	1.80
SERPINB2	9.15		4.59	0.69			2.34
PROS							

Supplementary Table 1: The expression profile of Vitamin D components from SARS-CoV2 infected patient samples when compared to its healthy controls and when the analysis performed in A549, NHBE and Calu3 cell lines

GO	Description	Count	%	Log10(P)	Log10(q)
COVID017	RNA_Blanco-Melo_Lung_Down	10	23	-11	-6.7
COVID255	RNA_Wilk_CD14+Monocytes_patient-C5_Down	5	12	-5.5	-1.5
COVID249	RNA_Wilk_CD14+Monocytes_patient-C2_Down	4	9.3	-5.5	-1.5
COVID352	RNA_Zhang_Monocytes_moderate-only_Up	3	7	-5.1	-1.3
COVID257	RNA_Wilk_CD14+Monocytes_patient-C6_Down	4	9.3	-5	-1.3
COVID011	RNA_Blanco-Melo_A549-ACE2-ruxolitinib_Down	5	12	-4.5	-1.1
COVID273	RNA_Wilk_Dendritic-cells_patient-C1A-mild_Up	3	7	-4.5	-1.1
COVID016	RNA_Blanco-Melo_Calu-3_Up	5	12	-4	-0.89
COVID032	RNA_Liao_BALF-severe_Up	5	12	-4	-0.89
COVID048	RNA_Wyler_Calu-3_12h_Up	5	12	-4	-0.89
COVID251	RNA_Wilk_CD14+Monocytes_patient-C3_Down	3	7	-3.9	-0.86
COVID252	RNA_Wilk_CD14+Monocytes_patient-C4_Up	3	7	-3.6	-0.63
COVID244	RNA_Wilk_CD14+Monocytes_patient-C1A-mild_Up	3	7	-3.6	-0.63
COVID253	RNA_Wilk_CD14+Monocytes_patient-C4_Down	3	7	-3.3	-0.46
COVID254	RNA_Wilk_CD14+Monocytes_patient-C5_Up	3	7	-3.2	-0.44
COVID209	Proteome_Li_Urine-recovery-vs-healthy_Down	3	7	-3	-0.32
COVID353	RNA_Zhang_Monocytes_severe-and-moderate_Up	3	7	-3	-0.32
COVID193	RNA_Stukalov_A549-ACE2_30h_Up	4	9.3	-3	-0.3
COVID051	RNA_Xiong_BALF_Down	4	9.3	-2.9	-0.3
COVID010	RNA_Blanco-Melo_A549-ACE2_Up	4	9.3	-2.9	-0.3

Supplementary Table 2: Results of oene ontology analysis of 43 genes specifically for COVID19 datasets using Metascape.

GO	Category	Description	Count	%	Log10(P)	Log10(q)
R-HSA-6798695	Reactome Gene Sets	Neutrophil degranulation	8	18.6	-6.22	-2.46
GO:0043900	GO Biological Processes	regulation of multi-organism process	7	16.3	-5.42	-2.12
GO:0043065	GO Biological Processes	positive regulation of apoptotic process	8	18.6	-5.07	-1.87
ko04142	KEGG Pathway	Lysosome	4	9.3	-4.42	-1.42
GO:0010951	GO Biological Processes	negative regulation of endopeptidase activity	5	11.6	-4.33	-1.39
hsa05169	KEGG Pathway	Epstein-Barr virus infection	5	11.6	-4.03	-1.18
GO:0007159	GO Biological Processes	leukocyte cell-cell adhesion	5	11.6	-3.63	-0.83
GO:0010959	GO Biological Processes	regulation of metal ion transport	5	11.6	-3.47	-0.71
GO:0051640	GO Biological Processes	organelle localization	6	14	-3.06	-0.44
GO:0008277	GO Biological Processes	regulation of G protein-coupled receptor signaling pathway	3	6.98	-2.75	-0.22
ko04621	KEGG Pathway	NOD-like receptor signaling pathway	3	6.98	-2.64	-0.15
GO:0007015	GO Biological Processes	actin filament organization	4	9.3	-2.35	0

Supplementary Table 3: Results of Gene Ontology analysis of 43 using Metascape.

	Pathway	Total	Expected	Hits	P.Value	FDR
1	Regulation of activated PAK-2p34 by proteasome mediated degradation	48	2.49	37	5.56E-39	7.79E-36
2	CDK-mediated phosphorylation and removal of Cdc6	49	2.54	37	2.17E-38	1.52E-35
3	Vpu mediated degradation of CD4	50	2.59	37	7.96E-38	3.72E-35
4	Stabilization of p53	54	2.8	38	1.92E-37	5.38E-35
5	Destabilization of mRNA by AUF1 (hnRNP D0)	54	2.8	38	1.92E-37	5.38E-35
6	Activation of NF-kappaB in B Cells	66	3.42	41	6.27E-37	1.47E-34
7	p53-Dependent G1/S DNA damage checkpoint	59	3.06	39	9.82E-37	1.72E-34
8	p53-Dependent G1 DNA Damage Response	59	3.06	39	9.82E-37	1.72E-34
9	Ubiquitin-dependent degradation of Cyclin D1	49	2.54	36	1.26E-36	1.77E-34
10	Ubiquitin-dependent degradation of Cyclin D	49	2.54	36	1.26E-36	1.77E-34
11	Autodegradation of the E3 ubiquitin ligase COP1	53	2.75	37	2.90E-36	3.69E-34
12	Degradation of beta-catenin by the destruction complex	65	3.37	40	8.43E-36	9.09E-34
13	Signaling by Wnt	65	3.37	40	8.43E-36	9.09E-34
14	G1/S DNA Damage Checkpoints	62	3.21	39	1.82E-35	1.83E-33
15	Ubiquitin Mediated Degradation of Phosphorylated Cdc25A	52	2.69	36	4.32E-35	3.57E-33
16	p53-Independent DNA Damage Response	52	2.69	36	4.32E-35	3.57E-33
17	p53-Independent G1/S DNA damage checkpoint	52	2.69	36	4.32E-35	3.57E-33
18	SCF-beta-TrCP mediated degradation of Emi1	53	2.75	36	1.29E-34	1.00E-32
19	Cross-presentation of soluble exogenous antigens (endosomes)	48	2.49	34	1.00E-33	7.14E-32
20	Vif-mediated degradation of APOBEC3G	55	2.85	36	1.02E-33	7.14E-32
21	Regulation of Apoptosis	59	3.06	37	1.32E-33	8.83E-32
22	Regulation of ornithine decarboxylase (ODC)	49	2.54	34	3.12E-33	1.99E-31
23	CDT1 association with the CDC6:ORC:origin complex	57	2.95	36	7.05E-33	4.30E-31
24	SCF(Skp2)-mediated degradation of p27/p21	58	3.01	36	1.77E-32	1.04E-30
25	ER-Phagosome pathway	63	3.26	37	4.38E-32	2.45E-30
26	Regulation of mRNA Stability by Proteins that Bind AU-rich Elements	88	4.56	42	2.52E-31	1.36E-29
27	Assembly of the pre-replicative complex	63	3.26	36	1.22E-30	6.35E-29
28	Switching of origins to a post-replicative state	69	3.58	37	4.38E-30	2.12E-28
29	Orc1 removal from chromatin	69	3.58	37	4.38E-30	2.12E-28
30	Cyclin E associated events during G1/S transition	65	3.37	36	5.71E-30	2.67E-28
31	Cyclin A:Cdk2-associated events at S phase entry	66	3.42	36	1.20E-29	5.42E-28
32	Removal of licensing factors from origins	71	3.68	37	1.77E-29	7.51E-28
33	Regulation of DNA replication	71	3.68	37	1.77E-29	7.51E-28
34	APC/C:Cdc20 mediated degradation of	71	3.68	36	3.81E-28	1.57E-26

	Securin					
35	Autodegradation of Cdh1 by Cdh1:APC/C	68	3.52	35	1.11E-27	4.45E-26
36	Antigen processing-Cross presentation	78	4.04	37	1.50E-27	5.84E-26
37	DNA Replication Pre-Initiation	80	4.15	37	4.78E-27	1.76E-25
38	M/G1 Transition	80	4.15	37	4.78E-27	1.76E-25
39	Apoptosis	158	8.19	50	5.44E-27	1.96E-25
40	Cdc20:Phospho-APC/C mediated degradation of Cyclin A	76	3.94	36	8.46E-27	2.83E-25
41	APC/C:Cdh1 mediated degradation of Cdc20 and other APC/C:Cdh1 targeted proteins in late mitosis/early G1	76	3.94	36	8.46E-27	2.83E-25
42	APC/C:Cdc20 mediated degradation of mitotic proteins	76	3.94	36	8.46E-27	2.83E-25
43	Activation of APC/C and APC/C:Cdc20 mediated degradation of mitotic proteins	77	3.99	36	1.52E-26	4.95E-25
44	Regulation of APC/C activators between G1/S and early anaphase	84	4.35	37	4.29E-26	1.37E-24
45	Downstream Signaling Events Of B Cell Receptor (BCR)	173	8.96	51	6.84E-26	2.13E-24
46	Metabolism of RNA	339	17.6	71	7.01E-26	2.14E-24
47	Metabolism of mRNA	317	16.4	68	2.07E-25	6.17E-24
48	APC/C-mediated degradation of cell cycle proteins	89	4.61	37	5.43E-25	1.55E-23
49	Regulation of mitotic cell cycle	89	4.61	37	5.43E-25	1.55E-23
50	Synthesis of DNA	95	4.92	38	6.60E-25	1.85E-23
51	Signaling by the B Cell Receptor (BCR)	199	10.3	53	1.54E-24	4.22E-23
52	Host Interactions of HIV factors	141	7.31	45	1.65E-24	4.44E-23
53	Disease	945	49	121	8.38E-24	2.22E-22
54	DNA Replication	102	5.29	38	1.42E-23	3.68E-22
55	G1/S Transition	113	5.86	38	1.01E-21	2.57E-20
56	Immune System	1140	59	131	1.31E-21	3.28E-20
57	S Phase	122	6.32	39	2.31E-21	5.69E-20
58	HIV Infection	214	11.1	51	3.05E-21	7.37E-20
59	Cell Cycle Checkpoints	131	6.79	40	4.66E-21	1.11E-19
60	M Phase	233	12.1	52	2.91E-20	6.80E-19
61	Separation of Sister Chromatids	186	9.64	46	6.47E-20	1.49E-18
62	Mitotic Anaphase	198	10.3	47	1.54E-19	3.48E-18
63	Mitotic Metaphase and Anaphase	199	10.3	47	1.93E-19	4.29E-18
64	Adaptive Immune System	654	33.9	90	2.88E-19	6.30E-18
65	Mitotic G1-G1/S phases	140	7.25	39	5.71E-19	1.23E-17
66	Mitotic M-M/G1 phases	266	13.8	53	2.77E-18	5.88E-17
67	Ribosomal scanning and start codon recognition	91	4.72	29	4.31E-16	9.03E-15
68	Translation initiation complex formation	92	4.77	29	6.01E-16	1.24E-14
69	Activation of the mRNA upon binding of the cap-binding complex and eIFs, and subsequent binding to 43S	93	4.82	29	8.33E-16	1.69E-14
70	Antigen processing: Ubiquitination & Proteasome degradation	224	11.6	45	9.32E-16	1.87E-14
71	Cell Cycle	508	26.3	71	1.91E-15	3.77E-14

72	Formation of the ternary complex, and subsequently, the 43S complex	83	4.3	27	2.60E-15	5.06E-14
73	Gene Expression	1090	56.3	112	2.10E-14	4.04E-13
74	Class I MHC mediated antigen processing & presentation	267	13.8	47	4.14E-14	7.84E-13
75	Cell Cycle, Mitotic	411	21.3	60	6.03E-14	1.13E-12
76	GTP hydrolysis and joining of the 60S ribosomal subunit	201	10.4	38	1.43E-12	2.57E-11
77	3' -UTR-mediated translational regulation	201	10.4	38	1.43E-12	2.57E-11
78	L13a-mediated translational silencing of Ceruloplasmin expression	201	10.4	38	1.43E-12	2.57E-11
79	Formation of a pool of free 40S subunits	189	9.79	36	4.61E-12	8.18E-11
80	Eukaryotic Translation Initiation	209	10.8	38	5.04E-12	8.72E-11
81	Cap-dependent Translation Initiation	209	10.8	38	5.04E-12	8.72E-11
82	Metabolism of amino acids and derivatives	190	9.85	36	5.42E-12	9.27E-11
83	Membrane Trafficking	203	10.5	37	9.09E-12	1.54E-10
84	TRAF6 Mediated Induction of proinflammatory cytokines	62	3.21	18	1.07E-09	1.79E-08
85	Translation	249	12.9	38	1.09E-09	1.79E-08
86	MyD88:Mal cascade initiated on plasma membrane	81	4.2	20	2.91E-09	4.59E-08
87	Toll Like Receptor TLR1:TLR2 Cascade	81	4.2	20	2.91E-09	4.59E-08
88	Toll Like Receptor TLR6:TLR2 Cascade	81	4.2	20	2.91E-09	4.59E-08
89	Toll Like Receptor 2 (TLR2) Cascade	81	4.2	20	2.91E-09	4.59E-08
90	Toll Like Receptor 10 (TLR10) Cascade	74	3.83	19	3.61E-09	5.50E-08
91	Toll Like Receptor 5 (TLR5) Cascade	74	3.83	19	3.61E-09	5.50E-08
92	MyD88 cascade initiated on plasma membrane	74	3.83	19	3.61E-09	5.50E-08
93	TRAF6 mediated induction of NFkB and MAP kinases upon TLR7/8 or 9 activation	76	3.94	19	5.85E-09	8.82E-08
94	Toll Like Receptor 7/8 (TLR7/8) Cascade	77	3.99	19	7.41E-09	1.09E-07
95	MyD88 dependent cascade initiated on endosome	77	3.99	19	7.41E-09	1.09E-07
96	Signal Transduction	1690	87.5	134	1.04E-08	1.51E-07
97	Toll Like Receptor 9 (TLR9) Cascade	79	4.09	19	1.17E-08	1.69E-07
98	Activated TLR4 signalling	100	5.18	21	2.63E-08	3.76E-07
99	Toll Like Receptor 4 (TLR4) Cascade	103	5.34	21	4.55E-08	6.44E-07
100	Fcgamma receptor (FCGR) dependent phagocytosis	86	4.46	19	5.18E-08	7.27E-07
101	TRIF-mediated TLR3/TLR4 signaling	87	4.51	19	6.32E-08	8.78E-07
102	Translocation of GLUT4 to the Plasma Membrane	71	3.68	17	7.54E-08	1.04E-06
103	MyD88-independent cascade	88	4.56	19	7.69E-08	1.04E-06
104	Toll Like Receptor 3 (TLR3) Cascade	88	4.56	19	7.69E-08	1.04E-06
105	Developmental Biology	417	21.6	48	8.85E-08	1.18E-06
106	Axon guidance	292	15.1	38	9.52E-08	1.26E-06
107	RIG-I/MDA5 mediated induction of IFN-alpha/beta pathways	67	3.47	16	1.91E-07	2.50E-06
108	Innate Immune System	521	27	54	4.29E-07	5.57E-06
109	MAP kinase activation in TLR cascade	55	2.85	14	4.85E-07	6.24E-06

110	Cytokine Signaling in Immune system	286	14.8	36	4.90E-07	6.25E-06
111	Platelet activation, signaling and aggregation	220	11.4	30	8.56E-07	1.08E-05
112	Toll-Like Receptors Cascades	123	6.37	21	1.06E-06	1.33E-05
113	NF-kB is activated and signals survival	13	0.674	7	1.24E-06	1.54E-05
114	Hemostasis	511	26.5	52	1.28E-06	1.58E-05
115	Recycling pathway of L1	44	2.28	12	1.45E-06	1.77E-05
116	Signaling by Interleukins	116	6.01	20	1.64E-06	1.98E-05
117	Signaling by EGFR in Cancer	181	9.38	26	1.76E-06	2.11E-05
118	Regulation of actin dynamics for phagocytic cup formation	62	3.21	14	2.32E-06	2.76E-05
119	Activation of the AP-1 family of transcription factors	10	0.518	6	3.26E-06	3.85E-05
120	Influenza Viral RNA Transcription and Replication	176	9.12	25	3.44E-06	3.99E-05

Supplementary Table 4: Functional enrichment analysis of 12 down regulated genes and its network using Reactome.

	Pathway	Total	Expected	Hits	P.Value	FDR
1	Immune System	1140	56.6	125	2.14E-20	3.00E-17
2	Adaptive Immune System	654	32.5	77	1.28E-13	8.99E-11
3	Cytokine Signaling in Immune system	286	14.2	44	8.44E-12	3.94E-09
4	Innate Immune System	521	25.9	62	3.39E-11	1.19E-08
5	NOTCH1 Intracellular Domain Regulates Transcription	50	2.49	17	1.02E-10	2.29E-08
6	Toll-Like Receptors Cascades	123	6.12	26	1.88E-10	2.29E-08
7	Constitutive Signaling by NOTCH1 HD+PEST Domain Mutants	52	2.59	17	2.07E-10	2.29E-08
8	Constitutive Signaling by NOTCH1 PEST Domain Mutants	59	2.93	18	2.20E-10	2.29E-08
9	Signaling by NOTCH1 t(7;9)(NOTCH1:M1580_K2555) Translocation Mutant	74	3.68	20	2.45E-10	2.29E-08
10	Signaling by NOTCH1 in Cancer	74	3.68	20	2.45E-10	2.29E-08
11	Signaling by NOTCH1 PEST Domain Mutants in Cancer	74	3.68	20	2.45E-10	2.29E-08
12	FBXW7 Mutants and NOTCH1 in Cancer	74	3.68	20	2.45E-10	2.29E-08
13	Signaling by NOTCH1 HD Domain Mutants in Cancer	74	3.68	20	2.45E-10	2.29E-08
14	Signaling by NOTCH1 HD+PEST Domain Mutants in Cancer	74	3.68	20	2.45E-10	2.29E-08
15	Signaling by NOTCH1	74	3.68	20	2.45E-10	2.29E-08
16	Activated TLR4 signalling	100	4.97	22	2.23E-09	1.96E-07
17	Toll Like Receptor 4 (TLR4) Cascade	103	5.12	22	4.03E-09	3.33E-07
18	TRIF-mediated TLR3/TLR4 signaling	87	4.33	20	5.44E-09	4.24E-07
19	MyD88-independent cascade	88	4.38	20	6.73E-09	4.72E-07
20	Toll Like Receptor 3 (TLR3) Cascade	88	4.38	20	6.73E-09	4.72E-07
21	Signalling by NGF	290	14.4	39	7.97E-09	5.32E-07
22	Cell Cycle	508	25.3	55	1.74E-08	1.11E-06
23	Hemostasis	511	25.4	55	2.14E-08	1.31E-06
24	Signaling by SCF-KIT	142	7.06	25	2.27E-08	1.33E-06
25	Signaling by NOTCH	95	4.72	20	2.72E-08	1.53E-06
26	Activation of BAD and translocation to mitochondria	17	0.845	9	2.85E-08	1.54E-06
27	Signaling by ERBB2	164	8.15	26	1.09E-07	5.47E-06
28	Intrinsic Pathway for Apoptosis	37	1.84	12	1.13E-07	5.47E-06
29	Cell Cycle, Mitotic	411	20.4	46	1.13E-07	5.47E-06
30	Signaling by Interleukins	116	5.77	21	1.92E-07	8.98E-06
31	Translocation of GLUT4 to the Plasma Membrane	71	3.53	16	2.56E-07	1.16E-05
32	Signaling by ERBB4	152	7.56	24	3.70E-07	1.62E-05
33	Factors involved in megakaryocyte development and platelet production	155	7.71	24	5.36E-07	2.28E-05
34	Signaling by FGFR in disease	178	8.85	26	5.75E-07	2.37E-05
35	TRAF6 mediated induction of NFkB and MAP kinases upon TLR7/8 or 9 activation	76	3.78	16	6.90E-07	2.77E-05
36	Toll Like Receptor 7/8 (TLR7/8) Cascade	77	3.83	16	8.33E-07	3.15E-05
37	MyD88 dependent cascade initiated on endosome	77	3.83	16	8.33E-07	3.15E-05

38	Activation of BH3-only proteins	24	1.19	9	1.12E-06	4.08E-05
39	MHC class II antigen presentation	118	5.87	20	1.13E-06	4.08E-05
40	Toll Like Receptor 9 (TLR9) Cascade	79	3.93	16	1.20E-06	4.08E-05
41	Signaling by TGF-beta Receptor Complex	70	3.48	15	1.22E-06	4.08E-05
42	Signaling by FGFR	162	8.06	24	1.22E-06	4.08E-05
43	Downstream signal transduction	163	8.1	24	1.37E-06	4.47E-05
44	SOS-mediated signalling	14	0.696	7	1.79E-06	5.70E-05
45	S Phase	122	6.07	20	1.95E-06	5.96E-05
46	Nucleotide-binding domain, leucine rich repeat containing receptor (NLR) signaling pathways	55	2.73	13	1.96E-06	5.96E-05
47	Toll Like Receptor 10 (TLR10) Cascade	74	3.68	15	2.57E-06	7.24E-05
48	Toll Like Receptor 5 (TLR5) Cascade	74	3.68	15	2.57E-06	7.24E-05
49	MyD88 cascade initiated on plasma membrane	74	3.68	15	2.57E-06	7.24E-05
50	G2/M Checkpoints	48	2.39	12	2.58E-06	7.24E-05
51	Signaling by EGFR in Cancer	181	9	25	2.74E-06	7.53E-05
52	Activation of ATR in response to replication stress	41	2.04	11	3.21E-06	8.49E-05
53	Kinesins	41	2.04	11	3.21E-06	8.49E-05
54	Mitotic G2-G2/M phases	105	5.22	18	3.33E-06	8.63E-05
55	G0 and Early G1	27	1.34	9	3.53E-06	8.99E-05
56	RIG-I/MDA5 mediated induction of IFN-alpha/beta pathways	67	3.33	14	3.81E-06	9.55E-05
57	Disease	945	47	77	3.89E-06	9.57E-05
58	Mitotic G1-G1/S phases	140	6.96	21	4.71E-06	0.000114
59	Signaling by the B Cell Receptor (BCR)	199	9.89	26	4.94E-06	0.000117
60	DAP12 signaling	164	8.15	23	5.34E-06	0.000125
61	TAK1 activates NFkB by phosphorylation and activation of IKKs complex	22	1.09	8	5.95E-06	0.000137
62	Signaling by PDGF	189	9.4	25	6.05E-06	0.000137
63	TRAF6 Mediated Induction of proinflammatory cytokines	62	3.08	13	8.16E-06	0.000175
64	MyD88:Mal cascade initiated on plasma membrane	81	4.03	15	8.35E-06	0.000175
65	Toll Like Receptor TLR1:TLR2 Cascade	81	4.03	15	8.35E-06	0.000175
66	Toll Like Receptor TLR6:TLR2 Cascade	81	4.03	15	8.35E-06	0.000175
67	Toll Like Receptor 2 (TLR2) Cascade	81	4.03	15	8.35E-06	0.000175
68	G2/M Transition	102	5.07	17	9.25E-06	0.000191
69	NOD1/2 Signaling Pathway	38	1.89	10	1.10E-05	0.000223
70	Interferon gamma signaling	74	3.68	14	1.29E-05	0.000257
71	Downstream Signaling Events Of B Cell Receptor (BCR)	173	8.6	23	1.32E-05	0.000257
72	Interferon Signaling	173	8.6	23	1.32E-05	0.000257
73	TCR signaling	65	3.23	13	1.41E-05	0.000269
74	Downstream signaling of activated FGFR	150	7.46	21	1.42E-05	0.000269
75	p75 NTR receptor-mediated signalling	85	4.23	15	1.54E-05	0.000287
76	Downstream TCR signaling	48	2.39	11	1.68E-05	0.000308
77	Activation of the pre-replicative complex	32	1.59	9	1.69E-05	0.000308

78	DAI mediated induction of type I IFNs	13	0.646	6	1.84E-05	0.000327
79	AKT phosphorylates targets in the cytosol	13	0.646	6	1.84E-05	0.000327
80	Cytosolic sensors of pathogen-associated DNA	19	0.945	7	2.12E-05	0.000371
81	Cell Cycle Checkpoints	131	6.51	19	2.20E-05	0.000381
82	Signaling by EGFR	179	8.9	23	2.32E-05	0.000397
83	Chemokine receptors bind chemokines	59	2.93	12	2.53E-05	0.000427
84	Cyclin B2 mediated events	5	0.249	4	2.89E-05	0.000482
85	NGF signalling via TRKA from the plasma membrane	207	10.3	25	3.00E-05	0.000495
86	DAP12 interactions	182	9.05	23	3.04E-05	0.000496
87	GRB2 events in EGFR signaling	14	0.696	6	3.09E-05	0.000498
88	Apoptosis	158	7.86	21	3.17E-05	0.000505
89	IRS-related events triggered by IGF1R	91	4.52	15	3.57E-05	0.000562
90	G1/S Transition	113	5.62	17	3.70E-05	0.000576
91	SHC1 events in EGFR signaling	15	0.746	6	4.93E-05	0.00076
92	Signaling by Type 1 Insulin-like Growth Factor 1 Receptor (IGF1R)	94	4.67	15	5.28E-05	0.000796
93	IGF1R signaling cascade	94	4.67	15	5.28E-05	0.000796
94	Interleukin-1 signaling	45	2.24	10	5.42E-05	0.000808
95	Centrosome maturation	86	4.28	14	7.49E-05	0.00107
96	Recruitment of mitotic centrosome proteins and complexes	86	4.28	14	7.49E-05	0.00107
97	G1/S-Specific Transcription	16	0.796	6	7.57E-05	0.00107
98	p75NTR signals via NF-kB	16	0.796	6	7.57E-05	0.00107
99	TRAF6 mediated NF-kB activation	16	0.796	6	7.57E-05	0.00107
100	Developmental Biology	417	20.7	39	8.15E-05	0.00114
101	IRS-mediated signalling	87	4.33	14	8.53E-05	0.00118
102	RIP-mediated NFkB activation via DAI	11	0.547	5	0.000106	0.00146
103	Interferon alpha/beta signaling	68	3.38	12	0.00011	0.00148
104	Constitutive PI3K/AKT Signaling in Cancer	89	4.43	14	0.00011	0.00148
105	Rap1 signalling	17	0.845	6	0.000112	0.0015
106	Mitotic M-M/G1 phases	266	13.2	28	0.000122	0.0016
107	Inhibition of TSC complex formation by PKB	3	0.149	3	0.000122	0.0016
108	IRS-related events	90	4.48	14	0.000124	0.00162
109	SHC1 events in ERBB2 signaling	25	1.24	7	0.000156	0.00199
110	IKK complex recruitment mediated by RIP1	25	1.24	7	0.000156	0.00199
111	Signal Transduction	1690	83.9	113	0.00016	0.00202
112	SHC-related events triggered by IGF1R	18	0.895	6	0.000161	0.00202
113	Transcriptional activity of SMAD2/SMAD3:SMAD4 heterotrimer	43	2.14	9	0.000209	0.0026
114	Insulin receptor signalling cascade	95	4.72	14	0.000225	0.00275
115	Signaling by constitutively active EGFR	19	0.945	6	0.000226	0.00275
116	E2F mediated regulation of DNA replication	35	1.74	8	0.000248	0.003
117	Downregulation of ERBB2:ERBB3 signaling	13	0.646	5	0.000273	0.00327
118	Post-chaperonin tubulin folding pathway	20	0.994	6	0.000309	0.00363

119	Loss of proteins required for interphase microtubule organizationÃ,Â from the centrosome	65	3.23	11	0.000311	0.00363
120	Loss of Nlp from mitotic centrosomes	65	3.23	11	0.000311	0.00363

Supplementary Table 5: Functional enrichment analysis of 19 up-regulated genes and its network using Reactome.

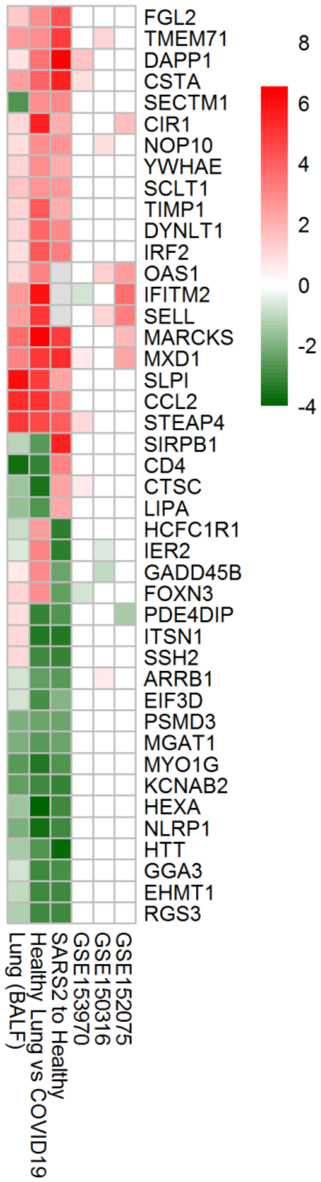

Supplementary Figure 1: Expression status of 43 share genes identified in Figure 3e in three independent validation datasets from SARS-CoV2 infected patient specimens. Expression levels of the 43 genes found differentially expressed in COVID19 patients were validated in other datasets from Gene expression Omnibus (Accessions: GSE153970, GSE150316, GSE152075). Genes followed the statistical thresholds as per Fig 3e, 15 out of 43 were observed as following the similar pattern of expression
